# Prediction of Protein-Protein Interaction Sites Based on Naive Bayes Classifier

**DOI:** 10.1155/2015/978193

**Published:** 2015-11-30

**Authors:** Haijiang Geng, Tao Lu, Xiao Lin, Yu Liu, Fangrong Yan

**Affiliations:** ^1^Research Center of Biostatistics and Computational Pharmacy, China Pharmaceutical University, Nanjing 210009, China; ^2^State Key Laboratory of Natural Medicines, School of Life Science and Technology, China Pharmaceutical University, Nanjing 210009, China

## Abstract

Protein functions through interactions with other proteins and biomolecules and these interactions occur on the so-called interface residues of the protein sequences. Identifying interface residues makes us better understand the biological mechanism of protein interaction. Meanwhile, information about the interface residues contributes to the understanding of metabolic, signal transduction networks and indicates directions in drug designing. In recent years, researchers have focused on developing new computational methods for predicting protein interface residues. Here we creatively used a 181-dimension protein sequence feature vector as input to the Naive Bayes Classifier- (NBC-) based method to predict interaction sites in protein-protein complexes interaction. The prediction of interaction sites in protein interactions is regarded as an amino acid residue binary classification problem by applying NBC with protein sequence features. Independent test results suggested that Naive Bayes Classifier-based method with the protein sequence features as input vectors performed well.

## 1. Introduction

A protein exerts its biological functions through interactions with other biomacromolecules, and these interactions occur on the residues of protein amino acid sequences. All the potential interaction sites, which the protein biochemical interactions occurred on, are on the surface of protein 3D conformation and called interface residues. Knowing the specific interface residues of proteins contributes to better understanding of protein-protein interaction mechanism. It is significant for researchers to be aware of interfaces residues in the study of protein mimetic engineering, molecular pathways elucidation, drug designing, and so on [1–3]. Determination of protein interaction sites in traditional experimental ways is detecting three-dimensional and crystal structure by NMR and X-ray, which is rather expensive and a consumption of time. Thus, there is a desperate need to develop new convenient and accurate computational ways of identifying protein-protein interface residues [4]. Newly discovered approach is utilizing all kinds of protein sequence and amino acid residue feature information to predict protein interfaces residues by using statistical classification methods.

The Protein Data Bank (PDB) [5] is a database containing biological molecules physical and structural data, submitted by biologists and biochemists from around the world. The increasing protein structures data in the PDB recently makes protein interaction sites prediction possible and available. Few recent computational methods of predicting the interface residue have been developed by using different features extracted from known protein interaction sites. Patch analysis [6] used a six-parameter function with chemical and physical characteristic features vectors of the known patches, such as flatness and hydrophobicity to predict interface patches. Other machine learning prediction methods include neural networks (ANN) [7–9], support vector machines (SVM) [10, 11], Conditional Random Fields (CFR) [12], Naive Bayes Classifier (NBC), and L1-Logistic Regression Classifier [13]. These methods applied structural, sequential, and evolutionary characteristic features of protein sequences, such as structural conservation score, amino acid residue composition, accessible surface area, residue evolutionary information, and spatial neighboring residues, as sequence features to distinguish interface residues and noninterface residues in protein sequences.

In this paper, we present the application of Naive Bayes Classifier (NBC) and used specific protein sequence features to distinguish the interface residues in protein-protein complexes. The NBC is a probability based machine learning algorithm and has been known to work efficiently for different classification tasks. So far, as reported, the NBC has been successfully used to predict the binding residues with DNA/RNA [14], the prediction of protein interaction partners [15], and the prediction of protein-protein interaction sites. Though NBC was a machine learning algorithm that already existed, different kinds of sequence features and input vector forms give rise to better or worse classification performance. The method in this paper used Position Specific Score Matrix (PSSM) and Relative Solvent Accessibility (RSA) as input feature vectors and was trained by a set of filtered protein amino acid residues with known interaction sites. Then, Leave One Out Cross Validation (LOOCV) was used to evaluate method performance. Independent test set showed that our classifier reached a sensitivity of 48.29%, a precision of 16.10%, a specificity of 62.11%, an accuracy of 60.30%, a MCC value of 7.71%, and an *F*-measure of 24.15%.

## 2. Methods

**Figure 1:**
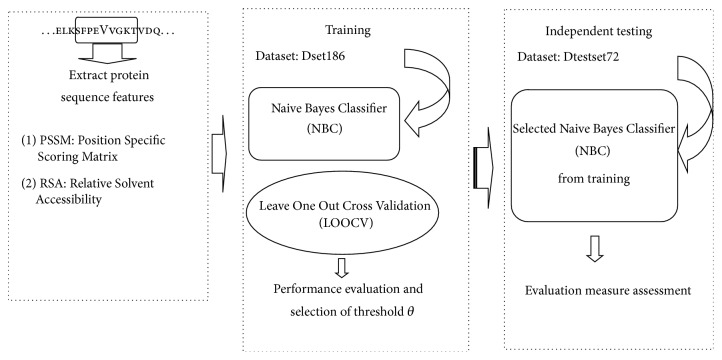
Schematic procedure outline of our study.

Naive Bayes Classifier in our method was trained on training set derived from protein sequence features; then, we used LOOCV results to evaluate model performance and the best model was selected. The NBC was also tested on independent test set. Schematic procedure outline of our study showed in Figure 1, details of datasets used for training and testing, definition of interface residues, Naive Bayes Classifier algorithm, and measurement of performance evaluation are mentioned in the following section.

### 2.1. Training Dataset

To obtain the training data of protein-protein complexes with two different chains used to develop a Naive Bayes Classifier, we extracted known biological dimeric protein-protein complexes in the PDB. To obtain a suitable nonredundant protein sequences dataset from PDB, we applied filtration conditions as follows:

1. Any proteins in PDB with a resolution of X-ray crystallography >3.0 Å or the protein sequence length less than 50 were excluded.
2. We used UniProt to filtrate heterodimers in reserved protein database. Proteins in the PDB were assigned with the UniProt accessions; remove any proteins constituted by two chains with the same UniProt accessions.
3. Missing ratio of a protein complexes is defined as missing residue number in a protein sequence/length of the sequence. Eliminate any protein complexes with a missing ratio of ≥30%.
4. Transmembrane proteins recorded in PDBTM were removed.
5. PDBsum was used to retain protein complexes with interface area between 500 Å^2^ and 2500 Å^2^.
6. Some of the remaining dimeric protein complexes, determined by above filters, that may be part of other protein complexes were also eliminated. These sequences would have different interaction sites while in different complexes.
7. The remaining sequences in the dataset conducted pair-wise clustering by BLASTClust. Eliminate sequences with a sequence identity of more than 25% from the dataset.

After the filtration of all the possible protein complexes in PDB, the finally obtained 186 different protein sequences composed the training data (Dset186) we used in this paper.

### 2.2. Testing Dataset

Independent test set is essential for any prediction method to confirm our method not only can be applied on the training data but also can be generally applied. We used protein-protein docking to obtain the independent test dataset. A set of protein complexes was obtained firstly; then BLASTClust was used to remove any sequences with sequence identity of more than 25% with sequences in Dset186. Protein sequences that are part of other complexes were not removed. After filtration, we obtained 72 different protein sequences (Dtestset72) extracted from 36 heterodimeric protein complexes.

### 2.3. Definition of Interface Residues

Definition details of surface residue and interface residue were as follows. Each residue in protein sequence was calculated with a Relative Solvent Accessibility (RSA) value; if its RSA was less than 5%, we defined it as a surface residue [16]. Moreover, we defined an interface if a surface one in complex formation lost its absolute solvent accessibility (SA) that exceeded 1.0 Å^2^ compared to that in the monomer formation. An amino acid residue was classified to be either interface class or noninterface. In this paper we used web server InterProSurf available at website http://curie.utmb.edu/pdbcomplex.html to determine the interface residue of protein complexes from its PDB number. Dset186 consists of 36219 residues of which 4241 (11.7%) were defined as interface residues and 2371 (13.1%) of 18140 residues in Dtestset72 were known as interface residues.

### 2.4. Naive Bayes Classifier

To predict the interface residues from a protein sequence, we trained a Naive Bayes Classifier. The NBC is machine learning classifier based on probability with assumptions that the features are independent from each other. According to the Bayes theorem, the conditional probability of a given residue classified into class *k* can be calculated as

(1) $$\begin{array}{c} p\left(\;C_{k}\;|\;X\;\right)=\frac{p\left(C_{k}\right)p\left(C_{k}\;|\;X\right)}{p\left(X\right)}. \end{array}$$

The training data used to generate the NBC has the expression formula of {*X*, *C*}. The sequence feature used to describe a residue is denoted by *X* = (*x*_1_, *x*_2_,…, *x*_*i*_,…, *x*_*n*_) and each residue belongs to a class *C* ∈ {0,1}, where 0 denotes a noninterface residue and 1 denotes interface. For a target residue with input *X*, the NBC conducts a binary classification by computing the posterior probability of a residue classified into a given class according to

(2) pC=1 ∣ X=x1,x2,…,xi,…,xn=1ZpC=1∏i=1npixi ∣ C=1,pC=0 ∣ X=x1,x2,…,xi,…,xn=1ZpC=0∏i=1npixi ∣ C=0.

Comparing the two posteriors according to (3) and taking logarithm according to (4),

(3) pC=1 ∣ X=x1,x2,…,xi,…,xnpC=2 ∣ X=x1,x2,…,xi,…,xn=pC=1∏i=1npixi ∣ C=1pC=0∏i=1npixi ∣ C=0,

(4) log⁡pC=1 ∣ X=x1,x2,…,xi,…,xnpC=2 ∣ X=x1,x2,…,xi,…,xn=log⁡pC=1∏i=1npixi ∣ C=1pC=0∏i=1npixi ∣ C=0.

The target residue represented by *X* was classified into interface class if

(5) $$\begin{array}{c} \log\frac{p\left(C=1\right)\prod_{i=1}^{n}p_{i}\left(x_{i}\;|\;C=1\right)}{p\left(C=0\right)\prod_{i=1}^{n}p_{i}\left(x_{i}\;|\;C=0\right)}≻\theta . \end{array}$$

Otherwise, *X* was deemed as noninterface residue and classified into class 0. In this paper *θ* was determined by the best result of the LOOCV.

### 2.5. Sequence Features

We incorporated the extracted sequence features Position Specific Score Matrix (PSSM) and Relative Solvent Accessibility (RSA) together as input vectors to NBC.

(1) Sequence features of the PSSM were calculated using PSI-BLAST [17]; parameters were set as follows: comparison database chosen NCBI nonredundant protein, *E*-value threshold 0.001, and iteration time 3. The PSSM represents evolutionary conservation information of a residue specific for its position in the protein chain. Interface residues are more conserved than noninterface surface residues [7]. In this paper, we used *p*(*i*, *n*) as the score value of an amino residue in the *n*th row of PSSM. Considering the neighbor effect of residues (discussed Section 3.1), we used a window size of 9 (containing 4 additional residues on each side) and the input vector was arranged from N-terminal side to C-terminal side with a subsequence of 9, as *X* = (*P*(*i* − 4,1),…, *p*(*i* − 4,20),…, *p*(*i*, 1), *p*(*i*, 20),…, *p*(*i* + 4,20)).
(2) As reported, interface residues always have higher solvent accessibility value than noninterface surface ones [9]. In protein three-dimensional complex formation noninterface residues do not have intermolecular forces; thus they lead to the decrease in solvent accessibility. The RSA of an amino acid residue is a real number that indicates the exposed solvent surface area. SABLE gives us the predicted real value of RSA for each residue, which ranges from 0 to 100.

A window is a subsequence of protein sequence with one central amino acid residue and same number of residues on either side. The window size means the number of residues in a subsequence. Here we used a window size of 9 and extracted a 181D (=20 × 9 + 1) feature vector, for each residue, either interface or noninterface. We also labeled every vector in the training data with 1 or 0, representing the interface or noninterface class they actually belong to. The window size was used for the consideration of neighborhood effect, which we will discuss later in this paper.

### 2.6. Evaluation Measures

The method was assessed according to the evaluation of prediction performance based on the following basic statistical results:

- TP means the number of predicted true positives residues, where actual interface residues are classified into interface class correctly.
- TN represents the number of predicted true negatives residues, where actual noninterface residues are correctly classified into noninterface class.
- FP means the number of predicted false positives residues, where actual noninterface residues are classified as interface residues incorrectly.
- FN represents the number of predicted false negative residues, where actual interface residues are incorrectly classified as noninterface residues.

The performance of the classifier was measured using Leave One Out Cross Validation (LOOCV). For each time, a different chain in the Dset186 was used as a test sequence and the rest as training data, repeated 186 times. Then we used the following measures to evaluate the classification performance:

(i) Sensitivity, sensitivity for interface residue class, measures the ratio of predicted interface to actual interface residues and is identified as TP/(TP + FN).
(ii) Precision, which measures the ratio of the predicted interface residues that are known as interface residues to the actual number of interface residues, is defined as TP/(TP + FP).
(iii) Specificity (SP) for the interface residue class measures the ratio of correctly predicted actual interface residues to all actual interface residues; SP is defined as TN/(TN + FP).
(iv) Accuracy (ACC) of a classifier measures the probability of correct prediction and is defined as (TP + TN)/(TP + FN + TN + FP); since the majority part of training data is noninterface class and the same with testing data, noninterface residues are much more likely to be predicted correctly; splendid high ACC value always means that the noninterface class predicted favorably; thus ACC is unsuitable to be the key measurement of the model performance.
(v) Matthews Correlation coefficient (MCC) is a measurement of how well the prediction results of interface residue class correlate with the actual interface residue class and MCC is defined as
  (6) $$\begin{array}{c} \frac{\left(\text{TP}\times \text{TN}\right)-\left(\text{FP}\times \text{FN}\right)}{\sqrt{\left(\text{TP}+\text{FP}\right)\times \left(\text{TP}+\text{FN}\right)\times \left(\text{TN}+\text{FP}\right)\times \left(\text{TN}+\text{FN}\right)}}. \end{array}$$
 The MCC value is generally considered as the most appropriate evaluation index for a prediction method [18]; the highest MCC value of 1 corresponds to the best performance that the method is able to classify all the interface residues correctly.
(vi) *F*-measure represents the harmonic mean of precision and sensitivity and the formula is defined as follows: 2 × (Precision × Sensitivity)/(Precision + Sensitivity).

## 3. Results

### 3.1. Interface Residues Tend to Cluster In Protein Amino Acid Sequence

**Figure 2:**
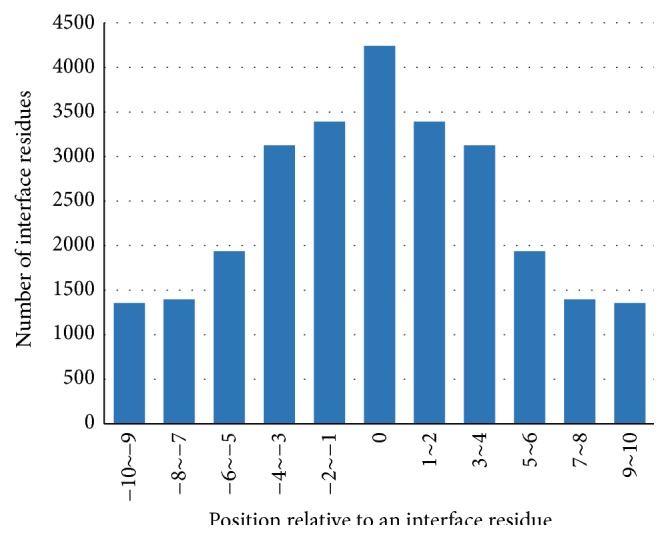
The number of neighboring interface residues for each position aside from an interface residue in Dset186. Position 0 is an interface residue and negative position represents the N-terminal side of this target residue and positive position is the C-terminal.

**Figure 3:**
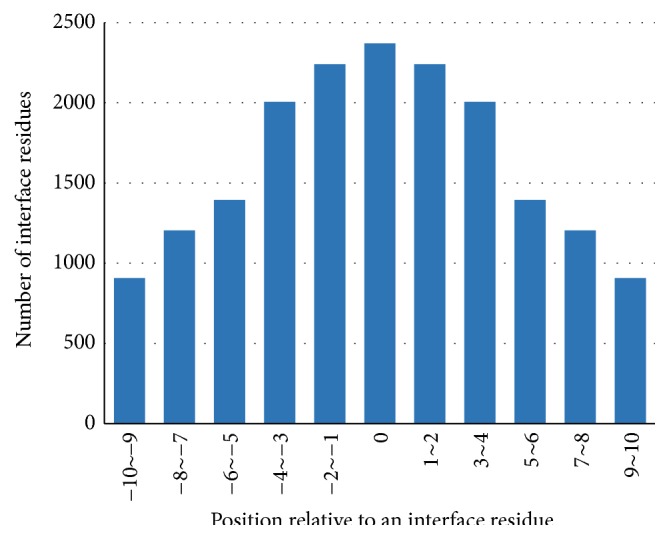
The number of neighboring interface residues for each position aside from an interface residue in Dtestset72.

To investigate the distribution of the known interface residues in protein sequences of the training and testing dataset, we calculated the number of neighboring interface residues for each position aside from the target residue from N-terminal side of an interface residue to C-terminal side and the results are shown in Figures 2 and 3. Then we observed the number of interface residues of each subsequence in window size of 3–11 coherent residues with the target interface residue on the central position and the results are shown in Table 1.

The number of neighboring interface residues aside from an interface residue observed in Figures 2 and 3 presents the pattern that this number decreases with the distance between the central interface residue. In Table 1, about 67%, 82%, 90%, 94%, and 95% of the interface residues have more than one actual interface residue in a window size of 3–11, respectively. Moreover, about 67.8% of the actual interface residues have more than three interface residues in window size 11 (with 5 residues on either side of the central interface residue). These results clearly indicate that interface residues have a tendency of clustering in protein sequences. We can know from Table 1 that there barely exists individual interface residue, but an interface residue tends to have additional interfaces residues in the neighborhood on protein sequence. Meanwhile, an actual interface residue affects the possibility that interface residues exist in the near neighbor. Thus a window or subsequence that contains a consecutive amino acid residues is used in predicting interface residue.

### 3.2. Model Selection

To determine which window size and threshold are the most suitable and perform the best, LOOCV was used for evaluation of model performance. We valued the window sizes of 3, 5, 7, 9, 11, and 13 and compared them with the situation that no window is used but only one residue is regarded as input feature; the threshold of each window size group ranges from −1 to 1, the best performance of each group was shown in Table 2. Compared with the other group, the results showed that the NBC with a window size of 9 and threshold of −0.88 has highest MCC and performs best.

**Figure 4:**
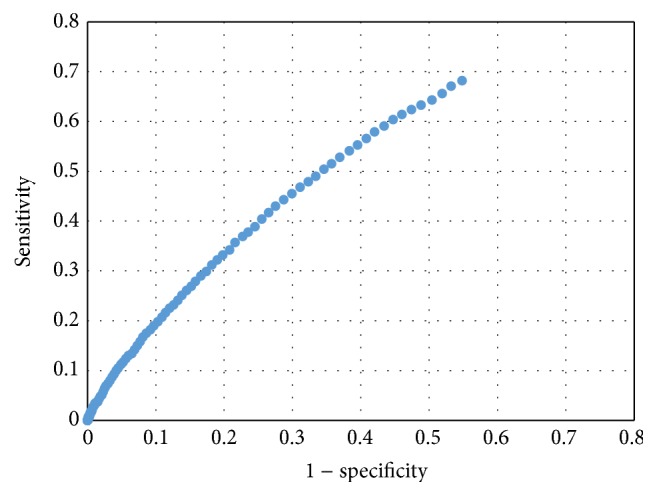
Dot plot of sensitivity versus specificity when NBC is with no window size.

The results also showed that threshold *θ* is the tradeoff between specificity and sensitivity. Specificity increases along with the growing of *θ* while sensitivity decreases. In some situations, where prediction model with high specificity is required, we can modify the threshold of NBC to make the specificity or sensitivity restricted to surpass a given valve.  Figure 4 shows the dot plot of sensitivity versus specificity when the NBC is with window size of 1.

### 3.3. Prediction Results and Comparison

The best performance of NBC model obtained above used a window size of 9 and threshold of −0.88. We trained the NBC on the condition of best performance above and the results of testing on independent Dtestset72 showed that the classifier reached a MCC value of 7.71%, an *F*-measure of 24.15%, a sensitivity of 48.29%, a precision of 16.10%, a specificity of 62.11%, and an accuracy of 60.30%. Meanwhile, there are other existing outstanding computational methods to predict interface residues, we compared our method with these reported prediction methods, including ISIS [19], SPPIDER [20], and PSIVER [21], which were tested on the same independent test set Dtestset72; Table 3 showed the best results of each model. Then, we also compared our model with several other machine learning algorithm methods, such as support vector machine (SVM), random forest (RF), and L1-regularized regression (L1RG). Trained with the same dataset and input vector structure, we applied these machine learning methods to test on independent Dtestset72; best performance (highest MCC value) of each algorithm was shown in Table 4.

MCC reveals the correlation coefficient between predicted interface residues and the actual interface residues and *F*-measure enumerates the harmonic mean of precision and sensitivity; both evaluate the overall performance of our method. Compared with the best performance of other reported methods tested on Dtestset72, the NBC has higher sensitivity value than ISIS, SPPIDER, and PSIVER, the MCC value is a little lower, and *F*-measure is about the same as the others. Moreover, an apparent merit of our method is that we have the highest sensitivity value, which means our method is more sensitive to the interface residues and more capable of identifying the actual interface residues. By comparing with other machine learning algorithms, our method showed an outstanding performance in sensitivity, MCC, and *F*-measure aspects.

## 4. Discussion

Developing accurate and valid computational methods to solve protein-protein interaction sites identification problem contributes to the mechanism study of protein function and benefits the researchers in drug designing. Methods that were developed to identify protein interface residues have been reported. In this paper we applied the Naive Bayes Classifier to predict the interface residues in protein complexes. The NBC was trained on Dset186 and also evaluated by LOOCV on Dset186. LOOCV results showed that the best performance reached a MCC value of 11.0%, an *F*-measure of 23.9%, an accuracy of 60.0%, a sensitivity of 56.9%, a precision of 15.2%, and a specificity of 60.4% with a window size of 9 and threshold of −0.88. Considering that interface residues tend to cluster in protein sequences and an actual interface residue affects the prediction of interface residue in its neighbor, we used window size as we input the protein sequence vectors.

Dset186 consists of totally 36219 amino residues of which 4241 residues were defined as interface; Dtestset72 contains 13213 residues of which 2510 residues were defined as interface. Obviously, both our training data and testing data are highly imbalanced datasets. The majority class in imbalanced datasets is always predicted favorably and high ACC value can be obtained easily. Nevertheless, the high ACC value did not contribute to improving model performance. Thus, Matthews Correlation Coefficient (MCC) becomes the most suitable evaluation index for the prediction of interface class. LOOCV results showed that our method shows a remarkable high MCC value of 0.11.

Independent test is of great importance and necessity for our method results to be persuasive and our model performed well in the independent test. In comparison with previous published methods, we used exactly the same test set Dtestset72 as ISIS, SPPIDER, and PSIVER to keep the comparison objective. Results showed that the NBC has higher sensitivity value than the other methods, the MCC value is a litter lower than the others, and the rest of the evaluation measures are basically the same. Since not all researchers used the same datasets, not all methods are publicly available, and different definition of interface residues varies among methods, we could not compare our method directly with many other reported methods. Moreover, the LOOCV results showed that our method performed better than the best LOOCV results of other extensive Naive Bayes-based method. Nevertheless, the independent test and comparison with ISIS, SPPIDER, and PSIVER indicate that out method is as feasible in practice as the other computational method and more capable in identifying the actual positive class as an interface residue. Then, the comparison with other machine learning algorithms showed in Table 4 indicates that our method performed extraordinarily better in sensitivity, MCC, and *F*-measure index.

In sequence feature selection, we used the combination of the two sequence features that has been previously predicted successfully, namely, position-specific scoring matrix (PSSM) and Relative Solvent Accessibility (RSA). PSSM was chosen as it represents the sequence conservation information and has been widely used in reported protein interface residues prediction methods. RSA was chosen as it was reported that it is more discriminating in classification process when using the relative value than the actual solvent accessibility alone. Based on the two kinds of sequence features we built the training data used to construct NBC.

For further application of our NBC-based method for identifying interface residues in protein complexes, we can use our method in actual experimental practice; the proposed method makes identifying the interface residues of an unknown protein more convenient and accurate for biologists. Compared with traditional ways, identifying interaction interface residues of unknown protein becomes more efficient and less expensive.

## Figures and Tables

**Table 1:** The ratio of actual interface residue number to subsequence length in different windows with an interface residue on the central position in the training dataset.

Window size	Ratio of actual interface residue number to subsequence length (%)										
	1	2	3	4	5	6	7	8	9	10	11
3	33.07	46.84	20.07								
5	18.08	32.14	27.04	16.52	6.19						
7	10.09	19.64	27.13	21.08	13.40	6.64	1.96				
9	6.57	11.93	20.89	21.72	17.68	11.93	6.07	2.46	0.66		
11	5.33	10.00	16.88	18.89	17.50	13.80	8.65	5.12	2.23	1.01	0.21

**Table 2:** The best LOOCV results of different window sizes for Dset186 among different threshold.

Window size	Sensitivity (%)	Precision (%)	Specificity (%)	ACC (%)	MCC (%)	*F*-measure (%)	Threshold θ
1	40.6	13.5	67.5	64.5	9.5	20.2	−1
3	53.1	14.5	60.9	60.0	8.9	22.7	−0.82
5	60.4	14.5	55.7	56.2	10.2	23.4	−0.98
7	54.3	15.1	62.2	61.3	10.5	23.7	−0.82
9	56.9	15.2	60.4	60.0	11.0	23.9	−0.88
11	56.0	15.1	60.8	60.3	10.7	23.8	−0.86
13	59.2	14.8	57.8	58.0	10.7	23.7	−0.96

**Table 3:** The best model performance of NBC, ISIS, SPPIDER, and PSIVER tested on Dtestset72.

Method	Sensitivity (%)	Precision (%)	Specificity (%)	ACC (%)	MCC (%)	*F*-measure (%)
NBC	48.3	16.1	62.1	60.3	7.7	24.2
ISIS	35.0	21.0	76.2	70.9	9.1	26.3
SPPIDER	45.4	20.4	64.7	61.7	8.1	24.6
PSIVER	46.5	25.0	69.3	66.1	13.5	27.8

**Table 4:** The best performance of machine learning algorithms tested on Dtestset72.

Method	Sensitivity (%)	Precision (%)	Specificity (%)	ACC (%)	MCC (%)	*F*-measure (%)
NBC	48.3	16.1	62.1	60.3	7.7	24.2
SVM	0.61	44.4	99.8	86.9	4.0	11.9
RF	2.5	19.5	98.4	85.9	2.5	4.5
L1RG	6.1	26.6	97.5	85.5	7.0	9.9
